# Children Treat Grammatical Errors Differently for Native and Non-Native Speakers

**DOI:** 10.3389/fpsyg.2022.855130

**Published:** 2022-04-22

**Authors:** Alexandra Rett, Katherine S. White

**Affiliations:** ^1^Department of Psychology, University of California, San Diego, La Jolla, CA, United States; ^2^Department of Psychology, University of Waterloo, Waterloo, ON, Canada

**Keywords:** accent-based expectations, foreign accents, grammatical errors, speaker reliability, speech errors

## Abstract

Both children and adults demonstrate biases against non-native speakers. However, in some situations, adults act more generously towards non-native speakers than towards native speakers. In particular, adults judge errors from non-native speakers less harshly, presumably because they expect such errors. In the present study, we asked whether 5-6-year-old children place less weight on errors from speakers with a foreign accent. In Experiment 1, 5- and 6-year-old children (*N* = 80) listened to pairs of either native or foreign-accented speakers (between-subjects) label objects. For native speaker pairings, children preferred information provided by grammatical speakers over information from speakers who made subject-verb agreement errors. In contrast, children chose between foreign-accented speakers at chance. In Experiment 2 (*N* = 40), children preferred information from grammatical foreign-accented speakers over information from foreign-accented speakers who produced word-order violations. These findings constitute the first demonstration that children treat speech errors differently based on a speaker’s language background.

## Introduction

Imagine that you are standing in front of a store window, looking at the objects inside. Someone approaches and exclaims, “Look, that are an amazing clock!”, while pointing at a clock on the wall. How will you react to this statement? It might depend on characteristics of the speaker. If the individual sounds like a native speaker of English, the grammatical error might capture your attention and cause you to consider other aspects of the speaker’s competence. However, you might react differently to the presence of such an error if the person is speaking English with a non-native accent. A growing body of literature has demonstrated that adults show more tolerance for errors produced by non-native speakers than errors produced by native speakers. However, the origins of these differences are not well understood, despite the fact that it is becoming increasingly common for people across the world to interact with speakers of other native languages. It is, therefore, important to understand when in development such accent-based expectations emerge and the experiences that give rise to them. In the present study, we take a first step in addressing these issues, by asking whether child listeners treat errors differently depending on who produces them.

Both children and adults show biases against non-native speakers in a variety of situations. For example, monolingual and bilingual children prefer native speakers over non-native speakers when they are pit against one another in friendship choice tasks (Kinzler et al., 2007; DeJesus et al., 2017; Paquette-Smith et al., 2019; Spence et al., 2021) and adults have more negative social evaluations of non-native speakers and the information they deliver (Gluszek and Dovidio, 2010; Lev-Ari and Keysar, 2010; Pantos and Perkins, 2013; Dragojevic and Giles, 2016; Baquiran and Nicoladis, 2020). However, adults have also been shown to be more forgiving towards non-native speakers (vs. native speakers) in some situations. For example, adults rate under-informative sentences as making more sense when they are told that the sentences were written by non-native speakers than when they are told that the same sentences came from native speakers (Fairchild and Papafragou, 2018). This appears to be driven by an assumption that non-native speakers produce “errors of omission” unintentionally, as a result of planning issues (Fairchild et al., 2020). Other work has also shown that adults’ neural and behavioral responses to errors produced by non-native speakers are attenuated. For example, Hanulíková et al. (2012) observed a typical neural response to grammatical errors (in this case, Dutch gender violations) when they were produced by a native Dutch speaker, but not when they were produced by a non-native (Turkish) speaker. And in some cases, adults internally correct errors produced by non-native speakers during processing (Gibson et al., 2017).

In the present study, we ask whether children treat grammatical errors differently for native vs. foreign-accented speakers. We do so by tapping into their judgments of speaker reliability. A large body of work on children’s judgments of informant reliability has shown that children take into account a variety of information when selecting an informant, such as their prior lexical accuracy (Koenig and Harris, 2005; Birch et al., 2008; Corriveau and Harris, 2009), confidence (Brosseau-Liard et al., 2014; Brosseau-Liard and Poulin-Dubois, 2014), age (Jaswal and Neely, 2006), accent (Kinzler et al., 2007, 2011; Howard et al., 2015; Petõ et al., 2018), and fluency (White et al., 2020). Not surprisingly, past accuracy appears to be the cue children rely on most strongly when evaluating information provided by others (Corriveau et al., 2013; Sobel and Finiasz, 2020). However, children excuse an individual’s past inaccuracy when there is a clear explanation for why they were wrong, such as not having access to necessary information (Nurmsoo and Robinson, 2009; Kondrad and Jaswal, 2012).

Here, we ask whether children treat inaccuracy (specifically, ungrammaticality) differently depending on an individual’s language background. Previous work has demonstrated that children endorse information provided by grammatical informants over ungrammatical informants when both are native speakers (Sobel and Macris, 2013). In that study, 4-year-olds were presented with two native-speaking informants who both produced lexically accurate statements, but differed in their use of syntax (one produced grammatical utterances and the other made subject-verb agreement errors). Older (but not younger) 4-year-olds subsequently chose to learn new object labels from the grammatical informant.

We predicted that children might treat the same kinds of grammatical errors differently when produced by foreign-accented speakers and, in particular, that they might place less weight on these grammatical errors. Recent work suggests that children can adjust their language processing based on information provided about individual speakers during the experimental session (e.g., Orena and White, 2015; Yurovsky et al., 2017). For example, although children use disfluencies (e.g., “um,” “uhh”) predictively in many situations (Kidd et al., 2011; Owens and Graham, 2016), they stop using them in this way when given reason to believe that disfluencies are uninformative for a particular speaker (e.g., someone forgetful; Orena and White, 2015). Although children have shown flexibility in processing for individual speakers, to our knowledge, there are no demonstrations that children show flexible interpretations of grammatical information based on a speaker’s linguistic group.

There at least two possible reasons why children might demonstrate differential treatment of grammatical errors from native- and foreign-accented speakers in our task. First, one explanation that has been offered for adults’ tolerance of errors from non-native speakers is that their previous experience interacting with non-native speakers leads them to expect errors. Indeed, some work has shown that these expectations can be quite specific. For example, although adults show an attenuation of the neural response to errors produced by non-native speakers (Hanulíková et al., 2012), this is not true across the board. When Spanish adults listen to English-accented Spanish, the neural response to gender violations (which are typical in non-native speech) is reduced, but the response to number violations (which are less typical) is not (Caffarra and Martin, 2019). Although it is not known how much experience is necessary to build up such expectations, it is entirely possible that children have at least a more general expectation that foreign-accented speakers will produce more grammatical errors, based on either their direct or indirect exposure to foreign-accented speech. In the real world, foreign accents do co-occur with grammatical differences from the native variety. Moreover, children are very sensitive to both accent (Kinzler et al., 2007) and grammatical violations (e.g., Santelmann and Jusczyk, 1998) from a young age. If children’s robust abilities to detect statistical patterns in their environment have enabled them to detect the relationship between foreign-accented speech and an increased frequency of grammatical errors, then they may not link such errors to other aspects of a speaker’s competence (such as their lexical knowledge).

Second, even if children do not have experience-based expectations about the grammaticality of different groups of speakers, they may still place less weight on errors produced by foreign-accented speakers for other reasons. One is that monolingual children appear to link foreign accents and errors to a similar cause. For example, when a native speaker makes speech errors, children use the presence of these errors to infer that the speaker is from “somewhere else” and to link them to non-local cultural items (Hwang and Markson, 2018). Children make similar inferences about speakers with unfamiliar accents and languages (Wagner et al., 2014; Weatherhead et al., 2018), suggesting that they associate both foreign accents and speech errors with unfamiliar or “far away” places. It is therefore possible that children could adjust their weighting of errors produced by foreign-accented speakers because they assume that the errors and accents stem from a common source. If so, they may assume that errors produced by foreign-accented speakers do not provide additional information about other aspects of their knowledge. This type of inference may not require any specific experience with foreign-accented speakers, but may instead reflect children’s assumptions that these characteristics both signal out-group membership.

To examine children’s treatment of errors in native vs. foreign-accented speech, we used the same type of subject-verb agreement errors used by Sobel and Macris (2013), but varied (across children) the accent of the informant who produced them. In Experiment 1, children listened to a grammatical (e.g., “that is a dog”) and an ungrammatical^1^ (e.g., “that am a shoe”) informant label familiar objects. Following this exposure, the two informants each labeled a different novel object using the same label and children were asked which object was the referent of the label. Children in the *native-accent* condition heard only speakers with a native English accent; children in the *foreign-accent* condition heard only speakers with a foreign accent. We expected that children in the native-accent condition would endorse the object referred to by a syntactically correct informant over a syntactically incorrect informant, replicating previous work. However, if children place less weight on grammatical errors for foreign-accented speakers, then they should show less influence of grammaticality when choosing between foreign-accented informants. Importantly, although children tend to endorse information provided by native speakers when native and foreign-accented speakers are pit against one another, children do sometimes endorse foreign-accented speakers in this type of reliability task. For example, Corriveau et al. (2013) demonstrated that if a foreign-accented speaker is observed to be somewhat more accurate than a native speaker in labeling familiar objects, children will endorse that foreign-accented speaker’s labels for novel objects. Therefore, if children do not selectively endorse the grammatical foreign-accented speakers here, it cannot simply be explained by an across-the-board failure to trust foreign-accented speakers.

## Experiment 1

### Method

#### Participants

Eighty 5- to 6-year-old children participated in the study. Children were randomly assigned to either the *native-accent* condition (n = 40, *M*_*age*_ = 6.1 years, range = 5.0 - 6.9 years, 19 females) or the *foreign-accent* condition (n = 40, *M*_*age*_ = 5.9 years, range = 5.1 - 6.9 years, 23 females). Sobel and Macris (2013) observed an effect of age in their study with 4-year-olds, such that older 4-year-olds were more likely to use grammaticality as a cue to speaker reliability than younger 4-year-olds. Therefore, in order to ensure that we would find robust endorsement of information provided by grammatical informants in our native-accent condition (to which we could compare performance in our foreign-accented condition), we tested 5-6-year-olds. The sample size was based on previous work examining children’s reliability judgments in this type of task. Most directly, it follows from the Sobel and Macris (2013) study, in which sample sizes of 32 and 39 per experiment were used^2^. Based on parent report, all participants were monolingual native English speakers who were not regularly exposed to a language other than English (i.e., less than 10% of language exposure consisted of another language). Data collection took place in children’s museums in Southern Ontario, Canada between May 2018 and September 2019. Most participants were tested in a region in which 75% of residents in the region identify English as their mother tongue, 1% report French and 24% report another language. Fifteen of the 80 participants were tested in an adjacent region in which up to 46% of residents report a non-English language as their mother tongue^3^. Seven additional children were tested but were excluded from analyses because of refusal to participate (2), significant exposure to, and proficiency in, a language other than English (1), experimenter error (3), and because the child claimed to be familiar with one of the novel objects and made their choice based on this familiarity (1). Parents provided written informed consent for all participants.

#### Materials

Four female speakers recorded the audio stimuli in a sound-treated recording booth using a MXL 770 Cardioid Condenser Microphone. Two of the speakers were native English speakers from Southern Ontario (the same region as the participants). Two of the speakers were late English learners whose native language was Chinese (one speaker’s native language was Cantonese while the other speaker’s native language was Mandarin), who spoke with perceptible foreign accents. They were judged by adult listeners to have non-native accents of moderate and equivalent strength. Children assigned to the native-accent condition heard only the native English speakers; children assigned to the foreign-accent condition heard only the foreign-accented speakers. Each speaker recorded a set of grammatical and ungrammatical sentences, which were used as the familiarization and test stimuli (described in detail below).

**FIGURE 1:**
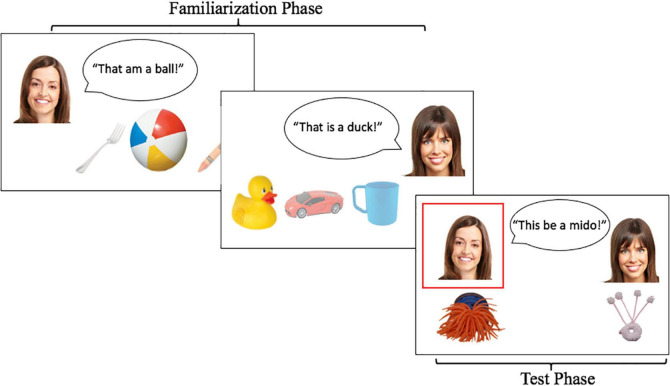
Example of familiarization and test phase of one trial of Experiment 1. Children first saw each informant label a set of familiar objects. This was followed by the test phase, in which each informant used a label to describe a novel object. A red box appeared around each informant as her test utterance played (Photo 13942088 / Smiles © Kurhan | Dreamstime.com).

The visual stimuli were identical for the native and foreign-accent conditions. On each trial, children saw two informant images, matched in attractiveness and hair color and type. Different images were used for the two trials (see Figure 1 for one speaker pairing). Within each condition (native-accent, foreign-accent), audio recordings from one speaker were used for the grammatical informant on both trials and audio from the second speaker was used for the ungrammatical informant on both trials. Across children, the assignment of speaker to informant grammaticality was counterbalanced, as was the pairing of speaker and informant image.

During familiarization, children saw the informant images along with images of familiar objects (6 familiar objects per trial, 3 per informant). The familiar objects were chosen to be items that 5- to 6-year-old children could easily label (e.g., a tree, a sock, an apple, a shoe, a dog and a spoon). On test trials, each informant was paired with a novel object (Figure 1). Novel object images were taken from the Novel Object and Unusual Name (NOUN) database (Horst and Hout, 2016).

#### Procedure

The experiment was presented to children on a 9.7-inch Apple iPad. Children were tested in a quiet location in the museum and were given headphones to wear for the duration of the study. Children wore headphones to ensure they could clearly hear the audio and so the experimenter was blind to informant status (i.e., the experimenter could not tell which informant was grammatical or ungrammatical).

##### Introduction Phase

To introduce the task, the experimenter told children that they would play a game in which they had to figure out the names of some new toys. Children were then shown images of two novel toys on the screen. The experimenter indicated that one of the two toys was called, e.g., a *mido*, and the child’s job was to figure out which toy it was. After introducing the novel toys, the experimenter introduced the two informants. The child was told that only one of the two informants knew which toy was a *mido*, and the child had to figure out which girl knew what toy it was. When the two informants were introduced in the foreign-accent condition, children listened to each informant say one of two short phrases. The first informant to be introduced said, “Hello, let’s play a game,” to which the second responded, “Hi there. Yes, let’s play!” These phrases were introduced prior to the familiarization phase so that children were aware of the fact that both informants had foreign accents before hearing the critical material. The length and content of the introductory phrases were designed so that they did not give children any information about either informant’s grammatical ability (specifically, no information about subject-verb agreement using the verb *to be*). Other than these added sentences, the procedure in the native- and foreign-accent conditions was identical.

##### Familiarization Phase

After introducing children to the task, each informant labeled three (different) familiar objects (Figure 1), with each object looming on the screen while it was being labeled. Following the procedure of Sobel and Macris (2013), both informants used the correct labels for the familiar objects. However, one used appropriate subject-verb agreement (e.g., when the image of a duck loomed, she said, “That is a duck.”) and the other used an incorrect form of the verb *to be* in their utterances. This ungrammatical informant used three different incorrect forms of the verb, one for each object in a trial. For example, when images of a ball, a fork, and a crayon appeared on screen, the ungrammatical informant said, “That am a ball,” “That be a fork,” and “That are a crayon.” It is important to note that this type of variability in errors, where the verb “to be” is replaced with a different alternative each time, is a somewhat extreme case. Although non-native speakers do exhibit variability in their error patterns (for example, using an incorrect determiner or no determiner at all in a particular context), this likely does not happen with the specific copula forms used here. However, if anything, this works against our hypothesis, as children might find these changing errors particularly salient, even for the foreign-accented speaker.

##### Test Phase

After exposure, children were presented with the test trial (Figure 1). The test trial contained two different question types: the endorse question and the explicit judgment question. The endorse question always occurred first and assessed which informant children thought was more reliable. Prior to the endorse question, the experimenter presented the child with the two novel objects again and reminded them that “one of these toys is a mido.” Each informant then labeled one of the two toys as a *mido*, and children were asked: “Which one of these is the mido?” While labeling the novel toys, each informant maintained the same grammatical structure as during exposure (i.e., the grammatical informant said, “This is a mido,” while the ungrammatical informant said, “This be a mido.”). This was done to reduce memory demands on children during the test. If children refused to respond or said, “I don’t know,” the experimenter repeated the endorse question or asked the child to point to the girl who labeled the correct toy. At the end of each trial, the experimenter asked children the explicit judgment question, which assessed children’s explicit awareness that the two informants differed in their grammaticality. The experimenter said, “One of these girls said silly things. Can you tell me which girl said silly things?” The same procedure was repeated for the second test trial (with different objects, informant images, and test labels).

### Results

Responses to the endorse question were coded as correct if children pointed to the toy labeled by the grammatical informant (or the grammatical informant themselves). Responses to the explicit judgment question were coded as correct if children pointed to the informant whose speech was ungrammatical (or toy indicated by that person). Correct responses were given a score of 1 and incorrect responses were given a score of 0. Analyses were conducted in R using the *geepack* package.

**FIGURE 2:**
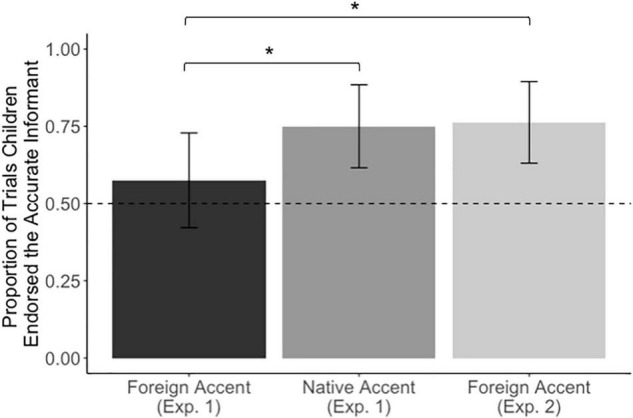
Proportion of trials in each experimental condition in which children endorsed the novel label provided by an accurate informant over an inaccurate (grammatically incorrect) informant. Error bars indicate 95% CI.

To examine if children’s judgments of reliability differed by condition, we used generalized estimating equations (GEE; independent correlation structure, binary logistic) to predict children’s informant choice (grammatical or ungrammatical) in response to the endorse question as a function of the following predictors: age (5 vs. 6 years), counterbalance, and condition (native-accent or foreign-accent condition). There were no main effects of age (χ^2^(1) = 1.40, *p* = 0.237) or counterbalance (χ^2^(1) = 2.39, *p* = 0.122). However, there was a significant main effect of condition (χ^2^(1) = 5.72, *p* = 0 .017, odds ratio = 2.17, 95% CI [1.15, 4.08]), as children in the native-accent condition were more likely to select grammatical informants (*M* = 75%, 95% CI [62%, 88%]) than children in the foreign-accent condition (*M* = 58%, 95% CI [42%, 73%]; see Figure 2).

To examine whether performance was different from chance in either condition, we used single-sample tests (intercept-only models) for each condition. These tests indicated that overall, children in the native-accent condition mostly selected grammatical informants in response to the endorse question, Wald (χ^2^(1) = 19.4, *p* <0.001), odds ratio = 3.00, 95% CI [1.8, 4.9], whereas the responses of children in the foreign-accent condition did not differ from chance, Wald (χ^2^(1) = 2.04, *p* = 0.150)^4^, odds ratio = 1.35, 95% CI [0.89, 2.0].

Next, we examined children’s responses to the explicit judgment question using GEE, predicting children’s informant choice (grammatical or ungrammatical) as a function of the same predictors (age, counterbalance, and condition). There were no main effects of age or counterbalance (*p*s > .364). Notably, there was also no effect of condition (χ^2^(1) = 1.12, *p* = 0.291, odds ratio = 1.43, 95% CI [0.74, 2.8]): children in the native-accent (*M* = 68%, 95% CI [54%, 83%]) and foreign-accent condition (*M* = 75%, 95% CI [62%, 88%]) were equally likely to select ungrammatical informants when asked to indicate the “silly” speaker. A single-sample test (using an intercept-only model) indicated that overall, children mostly selected ungrammatical informants in response to the explicit judgment question, Wald (χ^2^(1) = 29.9, *p* < 0.001), odds ratio = 2.51, 95% CI [1.8, 3.5].

### Discussion

The results of Experiment 1 suggest that children evaluate errors from native and foreign-accented speakers differently. When two individuals both spoke with a native English accent, 5- and 6-year-olds were more likely to endorse novel object labels from an individual who used appropriate subject-verb agreement compared to an ungrammatical informant, replicating previous findings (Sobel and Macris, 2013). However, children treated the same subject-verb agreement errors differently when they were produced by foreign-accented speakers. When selecting between grammatical and ungrammatical foreign-accented speakers, children chose between the two informants at chance. Importantly, this difference between the conditions was significant, suggesting that children placed less weight on grammatical errors when assessing the reliability of foreign-accented speakers.

But did children simply not detect the errors produced by the foreign-accented speakers? Critically, children’s explicit judgments of the speakers rule out this possibility. In the foreign-accent condition, children reliably reported that the ungrammatical informant said “silly” things when asked for an explicit judgment, the same pattern as observed in the native-accent condition. This demonstrates that children’s failure to use grammatical accuracy as a cue to the reliability of foreign-accented speakers is not because of a difficulty *perceiving* the syntactic violations. This dissociation is similar to one observed by Gibson et al. (2013), who found that, although adult participants mentally corrected implausible sentences during a comprehension task, they were nevertheless able to accurately transcribe those same sentences in a transcription task.

In Experiment 2, we present children with foreign-accented speakers who produce more severe grammatical errors involving word order. This experiment allows us to address two issues. First, it allows us to rule out the possibility that children failed to endorse the grammatical foreign-accented speaker in Experiment 1 simply because both were determined to be unreliable. We deem this possibility unlikely, given that children have been shown to endorse information from foreign-accented speakers in similar tasks (e.g., Corriveau et al., 2013). However, if children endorse the grammatical foreign-accented speaker in Experiment 2, it will provide further evidence against this interpretation. Second, if children selectively endorse the grammatical foreign-accented speaker when the alternative speaker produces more severe grammatical errors, this will indicate that children are not indiscriminate in their treatment of errors from foreign-accented speakers. Instead, this would suggest that, although they may place less weight on grammatical errors for foreign-accented speakers, these errors do affect their judgments of speakers once they exceed some threshold. We do not include native-accented speakers in this experiment, given these goals (and the fact that in both Experiment 1 and Sobel and Macris, 2013, children used more minor grammatical errors when evaluating the reliability of native speakers).

## Experiment 2

In Experiment 2, we again presented children with a grammatical informant and an ungrammatical informant. However, this time the ungrammatical informant produced word-order violations (e.g., “That a cup blue is”). This type of grammatical error differs from the subject-verb agreement errors used in Experiment 1 in that word-order violations may have more severe consequences for processing than subject-verb agreement errors, at least in a language like English, where word order is critical for interpretation. Word order is a robust and early acquired aspect of the grammatical system of English (Brown, 1973; Gertner et al., 2006), and word order violations are easily detected by both adults and children (Wulfeck, 1993; Matthews et al., 2005; MacDonald, 2008). Although word-order violations are also produced less frequently by non-native English speakers than subject-verb agreement errors (Johnson and Newport, 1989), whether children are sensitive to the differing frequency of errors in foreign-accented speech is likely to be a function of their particular language experience. Regardless of their familiarity with the relative frequency of these types of errors, however, we predict that children’s choices between foreign-accented informants will be more influenced by the larger word-order violations of Experiment 2.

### Method

#### Participants

A new sample of 40 5- to 6-year-old children participated in Experiment 2 (*M*_*age*_ = 5.9 years, range = 5.0 – 6.9 years, 22 females). Based on parent report, all participants were monolingual, native English speakers who were not regularly exposed to a language other than English (i.e., less than 10% of language exposure during a regular week consisted of another language). Data collection took place in local children’s museums in Southern Ontario, Canada between January 2019 and September 2019. Seven additional children were tested but were excluded due to experimenter error (6) or significant exposure to, and proficiency in, a language other than English (1).

#### Materials & Procedure

The same two female, foreign-accented speakers from Experiment 1 recorded audio stimuli for Experiment 2. The materials and procedure used in Experiment 2 were identical to those used in Experiment 1, with the exception of the errors present in the descriptions of the objects and the addition of an adjective to each phrase describing a familiar object (see Table 1). Adjectives were added to make it possible to create three different types of phrases containing word-order violations (see Appendix for complete list of phrases used in Experiment 2).

**TABLE 1:** Example of errors (word-order violations, right column) produced by the ungrammatical speaker in Experiment 2 during familiarization.

Object	Grammatical	Ungrammatical (Word-Order Violation)
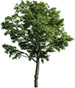	“That is a tall tree”	“That is a tree tall” OR “That a tall tree is” OR “That a tall is tree”
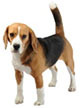	“That is a friendly dog”	“That is a dog friendly” OR “That a friendly dog is” OR “That a friendly is dog”
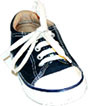	“That is a little shoe”	“That is a shoe little” OR “That a little shoe is” OR “That a little is shoe”

*Each type of error was used once on each trial.*

As in Experiment 1, in each trial, one informant produced grammatically correct utterances (e.g., when the image of a duck loomed, she said, “That is a yellow duck”) and one produced ungrammatical utterances. In Experiment 2, the ungrammatical informant produced three different word order errors, one for each familiar object in a trial (see Table 1). One error per trial always involved the incorrect ordering of the adjective and noun (e.g., “That is a tree tall”), and the other two errors involved the incorrect placement of the copula, *to be* (e.g., “That a friendly dog is,” “That a little is shoe”).

As in Experiment 1, while labeling the novel toys during the test, each informant maintained their grammatical status. Thus, in Experiment 2, the grammatical informant labeled the novel object by saying, “This is a mido,” while the ungrammatical informant said, “This a mido is.” Other than the change to the nature of the errors produced, the procedure of Experiment 2 was identical to that of the foreign-accent condition in Experiment 1.

### Results

Participants’ responses during the test phase were examined and coded in the same way as Experiment 1. Responses to the endorse question were coded as correct if children pointed to the toy labeled by the grammatical informant (or the individual herself) and responses to the explicit judgment question were coded as correct if children pointed to the informant whose speech was ungrammatical (or the toy that informant labeled). Correct responses were given a score of 1 and incorrect responses were given a score of 0. Analyses were conducted in R using the *geepack* package.

To examine if children selectively endorsed the object labeled by the grammatical informant, we again used GEE to predict children’s informant choice (grammatical or ungrammatical) in response to the endorse question (and separately in response to the explicit judgment question) using single-sample tests (intercept-only models). Condition was not included in this analysis, as there was only a single group of participants in Experiment 2. Overall, children mostly selected the grammatical informants (*M* = 77.5%, 95% CI [65%, 90%]) in response to the endorse question, Wald (χ^2^(1) = 15, *p* < .001), odds ratio = 3.44, 95% CI [1.8, 6.5]. To examine whether children selected the ungrammatical informant as the one who said “silly” things, we conducted the same analysis for the explicit judgment question (*M* = 72.5%, 95% CI [59%, 86%]). In response to this question, children mostly selected the ungrammatical informants, Wald (χ^2^(1) = 12, *p* < 0.001), odds ratio = 2.64, 95% CI [1.5, 4.6]. There were no effects of age or counterbalance (*p*s > 0.24).

#### Comparison of Experiments 1 and 2

To explore whether children’s endorsement of the grammatical, foreign-accented speaker differed across Experiment 1 and Experiment 2, we also used GEE (independent correlation structure, binary logistic) to predict children’s informant choice (grammatical or ungrammatical) in response to the endorse question as a function of the following predictors: age, counterbalance, and error type (the “agreement” errors in Experiment 1 or the “word-order” errors in Experiment 2). There were no main effects of age or counterbalance (*p*s > 0.674). However, there was a significant main effect of error type (χ^2^(1) = 6.11, *p* = 0.013), as children who heard word-order errors were more likely to select grammatical informants (*M* = 77.5%, 95% CI [65%, 90%]) than children who were exposed to agreement errors (*M* = 58%, 95% CI [42%, 73%]).

Finally, we examined children’s responses to the explicit judgment question when they heard foreign-accented speakers who produced either subject-verb agreement or word-order errors. There were no main effects of age or counterbalance (*p*s > 0.337). Notably, there was also no effect of error type, (χ^2^(1) = 0.106, *p* = 0.745): children who heard word-order errors (*M* = 72.5%, 95% CI [59%, 86%]) or subject-verb agreement errors (*M* = 75%, 95% CI [62%, 88%]) were equally likely to select ungrammatical informants when asked to indicate the “silly” speaker. A single-sample test (using an intercept-only model) indicated that overall, for both error types, children mostly selected ungrammatical informants in response to the explicit judgment question, Wald (χ^2^(1) = 30.5, *p* < 0.001).

### Discussion

The results of Experiment 2 demonstrate that monolingual children are not indiscriminate in their response to errors produced by foreign-accented speakers. When one foreign-accented speaker produced relatively severe grammatical errors (i.e., word-order violations), children were more likely to endorse a second, grammatical, foreign-accented speaker. This differs from their treatment of subject-verb agreement errors produced by foreign-accented speakers in Experiment 1. Thus, although children detected the errors from foreign-accented speakers in both experiments (as evidenced by their responses to the explicit judgment question), their use of these errors in the evaluation of speaker reliability was dependent on the nature of the errors produced.

## General Discussion

Previous work has demonstrated that children prefer to learn new words from an individual who has not made grammatical errors (Sobel and Macris, 2013). The present study demonstrates that children’s use of grammatical errors in the speech signal is influenced by a speaker’s accent. In Experiment 1, we employed the same types of grammatical errors used by Sobel and Macris (2013), but compared children’s treatment of native and foreign-accented speakers. Children were significantly more likely to endorse a novel label produced by a grammatical informant when they were listening to two native-accented speakers than when they were listening to two foreign-accented speakers. When two speakers had similar foreign accents, children’s judgments were dependent on the nature of the grammatical errors produced. Children were significantly more likely to endorse a grammatical speaker when the ungrammatical speaker produced word-order violations (Experiment 2) than when she produced subject-verb agreement errors (Experiment 1). Importantly, in all conditions of the present study (as in Sobel and Macris, 2013), informants used the correct labels for objects during familiarization. Therefore, both native and foreign-accented speakers demonstrated a typical level of lexical knowledge. Overall, these results demonstrate that children placed less weight on grammatical errors from foreign-accented speakers (Experiment 1), but that they did not ignore them completely (Experiment 2).

Our results suggest that children either do not treat minor grammatical errors as relevant in assessing a foreign-accented speaker’s competence overall or, more specifically, that they do not consider such minor errors to be linked to a foreign-accented speaker’s lexical knowledge. Importantly, both the results of Experiment 2 and previous research have suggested that children do not mis-trust foreign-accented speakers across the board. Thus, we believe that our results provide important insight into how children interpret errors and, to our knowledge, constitute the first demonstration that children’s interpretation of speech errors depends on the speaker’s language background.

### What Underlies Children’s Treatment of Errors From Foreign-Accented Speakers?

One explanation for adults’ differential processing of native and non-native speech is that they have experience-based expectations that non-native speakers will produce more errors. Moreover, there is evidence that at least some of these expectations involve specific types of errors. For example, when Spanish adults listen to English-accented Spanish, the neural response to gender violations (which are typical for non-native speakers) is reduced, but the response to number violations (which are less typical) is not (Caffarra and Martin, 2019).

It is not clear how much experience would be necessary for children to build up such expectations. We were not able to collect detailed information about our participants’ accent exposure and, although all of our participants were monolingual English speakers and were reported to have little exposure to languages other than English, many may very well have had exposure to speakers of English with various accents. If so, it is entirely plausible that they extracted a regularity that foreign accents co-occur with grammatical errors, given children’s sensitivity to statistical patterns in their input (see Saffran and Kirkham, 2018 for a review). While our study was not designed to measure or incorporate accent exposure, a future study should collect more extensive language exposure histories about participants, including the language proficiency of those they interact with regularly (since grammatical errors should be less frequent in foreign-accented speakers with higher proficiency). If children have indeed built up expectations from previous experience with foreign-accented speakers, then their performance in this type of task should be affected by their own experiences with foreign-accented speakers. Similarly, although we conceptualized the difference between the errors of Experiments 1 and 2 as being about error severity, future research could examine the effect of error severity vs. typicality. If children’s response is driven by typicality, rather than severity, we should again expect to see a relationship between children’s own language experiences and their treatment of different kinds of errors. We do note, however, that even our minor errors in Experiment 1 were not typical errors for non-native speech. Although non-native speakers can show variability in the realization of certain grammatical features in their second language, the variations do not tend to involve these specific variations in subject-copula agreement. Therefore, our results are consistent with the possibility that, even if children have developed expectations of foreign-accented speech, these expectations are not specific.

A second possibility is that children, despite not having had enough experience to extract a statistical regularity involving foreign accents and errors, may still link these two types of speech characteristics. Indeed, some work suggests that children use the presence of errors alone to infer that a speaker is from “far away” and to link them to non-local cultural items, inferences they also make about speakers with foreign accents (Hwang and Markson, 2018; Weatherhead et al., 2018). Although this is not direct evidence that children expect foreign-accented speakers to produce errors, it does suggest that they link a foreign accent and errors to a similar cause. If this link is at the root of children’s differential treatment of errors from native and foreign-accented speakers, then we may likewise see that children’s experience with foreign-accented speakers matters. However, the experience that would be relevant would be observing that foreign-accented speakers live in the same place as them, rather than experience with the particular features of their speech per se.

Finally, an intriguing possibility is that children’s different treatment of errors for the two types of speakers might have been triggered by features of the speech samples themselves. For example, some work has indicated that foreign-accented speech contains more variability at the phonetic and prosodic levels (Wade et al., 2007; Baese-Berk and Morrill, 2015). Children in our studies heard 5 sentences from each speaker prior to making their decision. If children detected increased variability in the sentences from our foreign-accented speakers, then they might have expected those individuals to be more variable in other ways as well. This could be a natural feature of processing - some work has suggested that differences in adults’ treatment of native and foreign-accented speech stem from signal-driven changes in the level of processing (Lev-Ari, 2015). Alternatively, children (even those with little exposure to other accents) could be generalizing from their experiences with another group of people who have more variable speech patterns, deviations from typical pronunciations, and grammatical errors – other children.

If the presence of heightened variability in the signal is what is driving children’s behavior, then children should respond differently when presented with speakers who have a different regional accent (e.g., speakers of Australian English). Although such speech would differ in many ways from children’s native variety (e.g., vowel pronunciation, prosody, etc.), it would contain no more variability, because it is produced by native speakers. If, instead, children are tracking the presence of pronunciations that are atypical for their native variety, then they should respond similarly to regional and foreign accents. Moreover, on either of these signal-based accounts, we might expect only a weak relationship between children’s performance and their specific patterns of previous exposure to foreign-accented speakers. We are currently investigating whether or not children attend differently to errors for speakers with regional and foreign accents.

### Implications for Children’s Flexible Use of Speech Information

Recent work has indicated that adult listeners are able to flexibly alter their weighting of different sources of information during language processing. For example, when the environment is noisy, listeners increase their use of visual speech cues or speaker-specific knowledge (Sumby and Pollack, 1954; Bejjanki et al., 2011). Similarly, adults’ expectations that non-native speakers are likely to make errors also appears to increase their reliance on contextual information or real-world knowledge for interpretation, as opposed to bottom-up information present in the speech signal itself (Lev-Ari, 2015; Gibson et al., 2017). This ability to flexibly alter the weighting of different sources of information is highly adaptive, allowing listeners to interpret language across noisy and uncertain environments.

While this evidence has accumulated in adults, there is only sparse evidence regarding whether or not children flexibly re-weight bottom-up and top-down information in this way during language comprehension. Yurovsky et al. (2017) demonstrated that children exposed to a single speaker in a noisy environment placed greater weight on their expectations of what the speaker likely intended to say than the acoustic signal. Our work does not show that children are less sensitive perceptually to grammatical errors from foreign-accented speakers, as they were equally likely to explicitly detect the errors for both native and foreign-accented speakers. Thus, for the types of errors used here, there is no evidence of attention being directed away from the speech signal. However, at a decision level, our results are consistent with the idea that they place less weight on the speech signal for foreign-accented speakers.

### Implications for How Children Attribute Knowledge to Others

Finally, our results also have important implications for understanding how children ascribe knowledge to others more generally. In particular, children do not appear to rigidly rely on the same cues to infer knowledge in all situations – even at the age of 5-6-years, children treat speech errors differently, depending on a speaker’s language background. In other words, they appear to reweight cues to a speaker’s knowledge given the context. This is perhaps surprising, as children are highly attuned to others’ accuracy, not just in reliability tasks such as this one (Koenig and Harris, 2005; Birch et al., 2008; Corriveau and Harris, 2009), but in other situations as well. In fact, children will even protest when newly learned words are applied inconsistently (Paulus and Worle, 2019). Although children do forgive past inaccuracy when there is an obvious reason for it (Nurmsoo and Robinson, 2009), in the present case, there was no such directly observable explanation for the inaccuracy.

The flexibility demonstrated here is consistent with suggestions that children’s reliability judgments reflect complex inferences about a speaker’s knowledge state, rather than simple associations (Southgate et al., 2010; Luchkina et al., 2018). In fact, the present results rule out an alternative associative explanation for Sobel and Macris (2013). In that study, the authors argue that children choose grammatical informants in a word learning task because they assume that an individual’s grammatical knowledge is informative about their lexical knowledge. However, in that study, children could simply have been making an association between the informant and errors (and carrying that over to the test phase). In the present study, children in the foreign accent condition of Experiment 1 did not choose an informant based on their grammaticality. A simple association account does not explain this finding. Instead, our results suggest that children do not obligatorily link grammatical and lexical knowledge. They make nuanced inferences, based on speaker identity, about what the grammatical errors indicate.

Our results further suggest that, in their evaluations of speaker reliability, children may be able to consider possible reasons for errors that are not based on visually obvious factors, such as an informant’s visual access to relevant information. If so, this study opens the door to using error “forgiveness” as a way of probing what specific or group level factors children view as relevant to an individual’s knowledge state. For example, might children also “forgive” errors from someone they are told is forgetful or who has difficulty with speech articulation? Might they “forgive” a child who makes an error in a situation in which they should not be expected to know it? In other words, just as children appear to view mouth obstruction as a possible explanation for atypical pronunciations (White and Daub, 2021), do they see certain characteristics of speakers as “explaining away” their errors? And, if they do, would they “forgive” only certain kinds of errors in these cases (those related to the individual’s specific or group level characteristics) or would they be overly generous in their forgiveness?

## Conclusion

The current findings suggest that children consider both speaker accent and error type when judging the reliability of individuals who produce grammatical errors. In particular, they appear to place less weight on errors produced by foreign-accented speakers when evaluating other aspects of their competence, such as their lexical knowledge. More work will be needed to determine what underlies these differences – whether they are a result of expectations that children bring to the task based on previous experience, whether they fall out of children’s other inferences about speakers of unfamiliar varieties, or whether they are instead generated in response to the speech signal itself. What we have shown, however, is that not all speech errors are judged in the same way: children’s interpretation of errors depends on who produced them.

## Data Availability Statement

The datasets presented in this study can be found in online repositories. The names of the repository/repositories and accession number(s) can be found below: https://osf.io/pm6f8/?view_only=694e4e10c46f4f59bcad00a5b74d45db.

## Ethics Statement

The studies were reviewed and approved by a Research Ethics Board at the University of Waterloo. Written informed consent to participate in this study was provided by the participants’ parents/guardians.

## Author Contributions

Both authors developed the concept, hypothesis, and design for the study. AR collected the data, conducted the analysis, and drafted the initial manuscript. KW supervised the study and revised the manuscript. Both authors contributed to the article and approved the submitted version.

## Conflict of Interest

The authors declare that the research was conducted in the absence of any commercial or financial relationships that could be construed as a potential conflict of interest.

## Publisher’s Note

All claims expressed in this article are solely those of the authors and do not necessarily represent those of their affiliated organizations, or those of the publisher, the editors and the reviewers. Any product that may be evaluated in this article, or claim that may be made by its manufacturer, is not guaranteed or endorsed by the publisher.

## Footnotes

1 Note that by grammatical “errors”, we refer to grammatical patterns that are not consistent with the listener’s dialect. The copula (Experiment 1) and word order (Experiment 2) changes used in the present study are ungrammatical in most varieties of English (with the exception of the invariant “be” construction used in AAE and some other American dialects), but it is not necessarily the case that patterns that are ungrammatical in one variety are ungrammatical in other varieties.

2 As our sample size was not pre-registered, we conducted an exploratory, post-hoc sensitivity analysis. A chi-squared test of the number of correct responses to the endorse question based on Condition revealed an effect size of w=0.23. Using the *pwr* package in R, we determined that an effect size of w = 0.31 could be reliably detected based on the parameters of our experiment (*N* = 80, alpha = 0.05, desired power = 0.80). Based on this, our study may have been underpowered. However, the observed effect size falls between what is considered to be a small (w = 0.10) or medium (w = 0.30) effect size (Cohen, 1988), and is above one recommended threshold for the minimum effect size representing a “practically” significant effect for social science data (Ferguson, 2009).

3 To account for potential differences based on the region in which participants were sampled, we conducted an exploratory analysis comparing performance on the endorse question between participants in the two regions. This revealed no effect of sampling location, χ^2^ (1) = 0.30, *p* = 0.60.

4 One possible concern pointed out to us by a reader of an initial draft is that children may have been responding to differences in the prosody across speaker conditions, rather than because of an expectation of errors in the foreign-accent condition. For example, the native speakers may have sounded more natural in the grammatical than ungrammatical sentences (leading them to endorse the grammatical speaker), whereas the foreign-accented speakers may have sounded equally unnatural in both (leading to no difference in endorsement). To address this possibility, we low-pass filtered the stimuli and collected naturalness judgments from 91 adults on Prolific. We found no relationship between naturalness judgments and children’s behavior in Experiment 1 or 2 that would be consistent with a prosody account.

## Appendix

**Appendix:** Phrases and Images Used in Experiment 2.

Object	Grammatical	Ungrammatical (Word-Order Violation)
		“That is a tree tall” OR
	That is a tall tree	“That a tall tree is” OR “That a tall is tree”
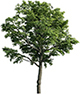

		“That is a sock colorful” OR
	That is a colorful sock	“That a colorful sock is” OR “That a colorful is sock”
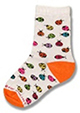

		“That is an apple red” OR
	That is a red apple	“That a red apple is” OR “That a red is apple”
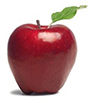

		“That is a shoe little” OR
	That is a little shoe	“That a little shoe is” OR “That a little is shoe”
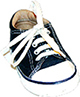

		“That is a dog friendly” OR
	That is a friendly dog	“That a friendly dog is” OR “That a friendly is dog”
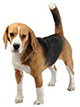

		“That is a spoon shiny” OR
	That is a shiny spoon	“That a shiny spoon is” OR “That a shiny is spoon”
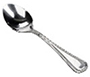

		“That is a crayon orange” OR
	That is an orange crayon	“That an orange crayon is” OR “That an orange is crayon”
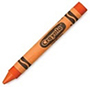

		“That is a fork silver” OR
	That is a silver fork	“That a silver fork is” OR “That a silver is fork”
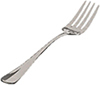

		“That is a ball round” OR
	That is a round ball	“That a round ball is” OR “That a round is ball”
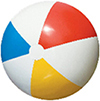

		“That is a duck yellow” OR
	That is a yellow duck	“That a yellow duck is” OR “That a yellow is duck”
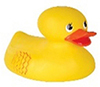

		“That is a car fast” OR
	That is a fast car	“That a fast car is” OR “That a fast is car”
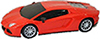

		“That is a cup blue” OR
	That is a blue cup	“That a blue cup is” OR “That a blue is cup”
